# Sustainability in the Public Healthcare Sector: Insights From an Analytical Hierarchy Process Analysis

**DOI:** 10.7759/cureus.61672

**Published:** 2024-06-04

**Authors:** Tariq Al Habsi, Salah Al-Khusaibi, Dalal Al Hashmi, Abdulrahman Al-Jabri, Adham Al-Rahbi, Nusaiba Almamari, Teeba Alkindi

**Affiliations:** 1 Department of Family Medicine and Public Health, College of Medicine and Health Sciences, Sultan Qaboos University, Muscat, OMN; 2 Department of Operation Management and Business Statistics, College of Economics and Political Sciences, Sultan Qaboos University, Muscat, OMN; 3 Department of Biology, College of Science, Sultan Qaboos University, Muscat, OMN; 4 Department of Biomedical Science, College of Medicine and Health Sciences, Sultan Qaboos University, Muscat, OMN; 5 Humanities Research Center, Sultan Qaboos University, Muscat, OMN

**Keywords:** oman, healthcare, environmental sustainability, sustainability, ahp, analytical hierarchy process, healthcare sustainability

## Abstract

Background

This study aimed to identify sustainability priorities within Oman’s healthcare sector using the analytical hierarchy process (AHP) methodology. Mainly, it focused on assessing the relative importance of economic, environmental, and social factors and their sub-elements in sustaining Oman’s healthcare system.

Methodology

A semi-quantitative, cross-sectional design was employed to collect data from 23 Omani healthcare experts with at least 10 years of experience in five different public hospitals in Oman. The AHP methodology was used to analyze pairwise comparisons of sustainability factors and derive their priorities. The consistency ratio was calculated to ensure the reliability of the analysis, and the transitivity rule was applied to address inconsistencies in pairwise comparisons.

Results

The findings revealed a strong emphasis on environmental and social sustainability, with economic considerations ranking comparatively lower. Circular practices emerged as a key priority within the environmental domain, while patient satisfaction stood out within the social domain. The analysis showed the integration between healthcare, environmental health, and societal well-being, emphasizing the need for holistic approaches to sustainability.

Conclusions

This study contributes to the understanding of healthcare sustainability by providing empirical evidence of stakeholder preferences within Oman’s healthcare system. By quantitatively assessing the relative importance of economic, environmental, and social factors, it informs decision-making and resource allocation toward sustainable healthcare development. The findings also support the argument for integrated approaches to healthcare sustainability that balance economic efficiency with environmental protection and social inclusion.

## Introduction

The first decade of the 21st century has witnessed strong growth in different sectors, especially in the economy, technology, and healthcare, which has created massive change. Therefore, governments must have the ability to evolve and keep pace with world requirements. If we highlight the healthcare sector, we will observe that it goes simultaneously with other sectors and reflects developments in the overall systems. The government would typically face many difficulties that have been hampering the promotion of development, such as a lack of resources in the healthcare system [1]. In Oman, the annual health report 2020 showed a higher expenditure rate of the Ministry of Health (MOH) compared to the previous three years (2017-2019) [2]. The report indicated that the total recurrent expenditures in MOH reached approximately 972.5 million Omani Riyal in 2020 compared to approximately 793.3 million Omani Riyal in 2019, representing a 22.6% increase [2]. These statistics of higher expenditures and a lack of resources stress the risk of long-term sustainability.

Healthcare sustainability can be considered a domain of quality, extending the responsibility of health services to patients not just for today but for the future. The long-term perspective highlights the impact of our healthcare system on our environment and communities, which, in turn, impacts population health. Yet, sustainability is a complex and challenging topic to assess [3]. Despite that, several studies have attempted to develop methods for its assessment, covering different dimensions of sustainability [4]. However, regarding measuring sustainability in healthcare, a few researchers have investigated this topic [5-7]. Thus, more research on healthcare sustainability approaches is required.

Due to the lack of such research in Oman, this study aims to define sustainability priorities in Omani healthcare using the analytical hierarchy process (AHP). The objectives of this study are to use an AHP-based model for healthcare sustainability measures and to understand the importance of these sustainability measures in Oman’s healthcare. Defining the priorities, in turn, will enable decision-makers to make better judgments, plans, and implementations toward sustainable development goals. Eventually, this is expected to contribute to Oman’s vision for 2040 by participating in achieving the objectives of the second national priority, health [8].

## Materials and methods

In this study, a semi-quantitative, cross-sectional design was employed to measure sustainability factors in Oman’s healthcare sector using the AHP method. Ethical approval was obtained for this study (reference number: 760-2023) before the conduction of the study. The sample size was determined for the present study by looking at the sample sizes of similar previous studies [5,7]. Using convenience sampling, 23 healthcare experts were included. The inclusion criteria were Omani professionals with at least 10 years of experience in healthcare management or clinical practice within Oman’s healthcare. Participants were recruited from the MOH and five different public hospitals in Oman. They were included based on their extensive experience or leadership roles within the healthcare sector in Oman. Individuals who did not meet the inclusion criteria, exclusively working in the private healthcare sector, or refused to participate were excluded from the study. As the AHP model focuses on expert opinions, such a small sample size is acceptable [9,10].

Data collection was performed between November 2023 and December 2023 through a healthcare sustainability measures questionnaire developed and validated by Mehra and Sharma [7]. The nine-point questionnaire was distributed using Google Forms to the experts to collect their opinions, along with consent forms. Clear instructions were given on how to fill out the questionnaire and participants’ questions were addressed. To ensure consistency and reliability in data collection, the definitions of each sustainability sub-element, as reported in a previous study [7], were included before their relevant questions. All responses were confidential throughout the data collection process.

After the consent form and the demographic data of the participants, the questionnaire was divided into four main sections. The first was a pairwise comparison between the three main sustainability elements, namely, economic, environmental, and social. The following three sections were a pairwise comparison between the sub-elements of each main element alone. The second section, for example, compared two environmental sub-elements each time ensuring that all environmental sub-elements were compared against each other. The same was followed for the other two sections. Thus, we had two different analysis levels. The first level focused on the main sustainability elements while the second level focused mainly on the sub-elements. Then, the AHP was used to analyze the answers and compare the two factors each time. It was selected to determine an overall ranking of all healthcare sustainability factors customized for the case of Omani healthcare to assist the decision-making process.

After collection, data were transferred to Microsoft Excel (Microsoft Corp., Redmond, WA, USA), and the analysis was done using the Analytic Hierarchy Process Software version 4.2.7. The mean of the participants’ responses was calculated. A pairwise comparison matrix was used to determine the weight of each element and sub-element. The consistency ratio (CR) was calculated to ensure the reliability of the analysis, and the transitivity rule was applied to address inconsistencies in pairwise comparisons. Eventually, the overall results were represented as tables and graphs.

## Results

The questionnaire was designed for healthcare experts to identify factors of sustainability in Omani healthcare, measure these sustainability factors, and prioritize their importance. The sample included 23 healthcare professionals. Male participants represented 65.2% (n = 15) of the study sample. More than half of the participants were senior directors/managers (60.9%, n = 14).

The values in the AHP questionnaire represent the pairwise comparisons of each factor against the others. Using a scale ranging from one to nine, the experts were asked to determine their opinion on the relative importance of each element over the other, where one indicated equal importance or priority and nine indicated the highest importance or priority.

The AHP analysis was conducted to assess the relative importance of the three sustainability elements, namely, economic, environmental, and social. Each of these elements had sub-elements [7], as presented in Table 1.

**Table 1:** Healthcare sustainability elements with their sub-elements. The explanations provided align with those established in the study by Mehra and Sharma [7] that published the questionnaire.

Elements	Sub-elements	Explanation
Economic	Green Growth (GG)	The incorporation of environmentally friendly practices into healthcare delivery, such as establishing a green hospital
	Indigenous Production (IPD)	The production from sources within a territory or a country
	Research and Innovations (RI)	The potential to contribute to fundamental changes toward sustainability, such as implementing improvement programs in clinical practice and nursing practice and reducing the cost of healthcare
	Savings in Operational Costs and Enhanced Profits (SOCEP)	Strategies that promote cost savings and profitability
Environmental	Facilities Design (FD)	The architectural design of a hospital facility, including its technology and equipment, and its effect on patient safety
	Circular Practices (CP)	The concept of keeping resources in use for as long as possible
	Sustainable Procurement (SP)	Combining social and environmental factors with financial considerations when making decisions based on life-cycle costs and associated environmental and social risks and benefits
	Waste Reduction & Management (WRM)	Reducing healthcare waste to the greatest extent possible, which is destined for ultimate disposal by means of reuse, recycling, and other programs
Social	Affordability (AF)	A function of income, spending, and judgments about the value of goods and services for their price
	Patient Satisfaction (PS)	The extent to which patients are satisfied with their healthcare services
	Sustainable Health (SH)	Focuses on the prevention of diseases and the promotion of healthy lifestyles
	Employee Satisfaction (ES)	When employees are happy and fulfilling their desires and needs at work

In the first stage, the experts were asked to compare the relative importance of the three main elements, i.e., economic, environmental, and social. Subsequently, for each of these elements, they were asked to compare the relative importance of their sub-elements. The final value was calculated from the geometric mean of the experts’ responses to the AHP questionnaire. After that, a pairwise comparison matrix was used to assign numerical values representing the preference or importance of one element over another. By normalizing the matrix, calculating the eigenvalues, and determining the principal eigenvector, the weights or priorities of the elements were derived. These weights indicate the relative significance of each element in the decision-making process. The pairwise comparison matrix for the main three elements before applying the transitivity rule is presented in Table 2.

**Table 2:** Pairwise comparison matrix for the main sustainability elements before and after applying the transitivity rule.

Main elements	Economic	Environmental	Social	Priorities
Before applying the transitivity rule				
Economic	1	0.1383	0.1333	0.0524
Environmental	72.306	1	73.904	0.7480
Social	74.993	0.1353	1	0.1996
After applying the transitivity rule				
Economic	1	0.1429	0.1428	0.0667
Environmental	7	1	1	0.4667
Social	7	1	1	0.4667

Then, the CR was calculated for the three main elements. The CR was 0.46045, which is higher than the acceptable threshold as the maximum acceptable threshold is 0.1. For this reason, the transitivity rule was applied to this matrix. The transitivity rule states that if there is a set of criteria or alternatives and pairwise comparisons between them, the resulting judgments should be transitive. In other words, if A is preferred over B, and B is preferred over C, then A should be preferred over C. By applying the transitivity rule, the pairwise comparison matrix was updated, as shown in Table 2.

Based on the analysis of the AHP pairwise comparison matrix, the following interpretations can be made. The priorities column represents the derived weights or priorities for each criterion based on the AHP analysis. The criterion with the highest priority has the highest weight, while the criterion with the lowest priority has the lowest weight. Based on these properties, the importance of the elements can be ordered from the lowest to the highest as economic, then environmental, and social together. The weight or priority of the economic element is 0.067. It has the lowest weight among the three elements, indicating that economic considerations are considered relatively less important compared to environmental and social factors. The economic element has only 6.7% of the overall priority. The weight or priority of both the environmental and social elements is 0.467. They have the highest ranking among the three elements in the decision-making process. They are seven times more important than the economic factor, with each representing 46.7% of the overall priority.

Next, the AHP analysis was done for the sub-elements of each of the three elements to determine the relative importance of each of them to the others. Starting with the pairwise comparisons of the economic sub-elements, the CR was also higher than the threshold (CR = 0.4355). Therefore, the transitivity rule was also assumed for the economic sub-elements. In this case, the pairwise comparison matrix was updated. Table 3 shows the pairwise comparisons of the economic sub-elements before and after applying the transitivity rule.

**Table 3:** Pairwise comparison of economic sub-elements before and after applying the transitivity rule. GG: Green Growth; RI: Research and Innovations; SOCEP: Savings in Operational Costs and Enhanced Profits; IPD: Indigenous Production

Economic sub-elements	GG	RI	SOCEP	IPD	Priorities
Before applying the transitivity rule					
GG	1	69.446	65.741	59.952	0.6191
RI	0.14399	1	77.219	74.453	0.2541
SOCEP	0.15211	0.1295	1	69.165	0.0906
IPD	0.16679	0.1343	0.1446	1	0.0362
After applying the transitivity rule					
GG	1	7	7	6	0.6885
RI	0.1429	1	1	0.8571	0.0983
SOCEP	0.1429	1	1	0.8571	0.0983
IPD	0.1667	1.1667	1.1667	1	0.1148

These sub-elements are Green Growth, Research and Innovations, Savings in Operational Costs and Enhanced Profits, and Indigenous Production. The values in the table indicate the relative importance or preference of one criterion over another. The priorities column represents the derived weights or priorities for each criterion within the economic element. Green Growth is considered the highest priority, as it has the highest value for its group. It is also given relatively high importance in comparison to the other factors, as it represents about 69% of the final decision. Green growth is considered seven times more important than both Research and Innovations and Savings in Operational Costs and Enhanced Profits. It is six times more important than Indigenous Production.

The Research and Innovations sub-element is considered the least important priority as it represents about 9.8% of the final decision. It shares equal priority with Savings in Operational Costs and Enhanced Profits in economic growth and sustainability decisions. Savings in Operational Costs and Enhanced Profits also represent less than 9.8% importance in the final economic growth and sustainability decision. It is considered one of the two least important factors in economic growth and sustainability decisions. Indigenous Production is the second-most important factor among all economic factors considered, representing 11% of the economic decision.

Table 4 shows the pairwise comparison matrix of the environmental sub-elements with and without applying the transitivity rule, as the CR is 0.29985, which is higher than the threshold. The sub-elements included are Circular Practices, Facility Design, Waste Reduction and Management, and Sustainable Procurement. Circular Practices are considered the highest priority in the environmental element. It represents about 67.7% of the environmental decision. It is six times more important than Facility Design and Waste Reduction and Management, and seven times more important than Sustainable Procurement. Facility Design and Waste Reduction and Management are both of the same priority. Each of them represents about 11% of the environmental element. Sustainable Procurement is given the least importance among all elements of the environmental criteria, representing 9.7% of the environmental decision.

**Table 4:** Pairwise comparisons of environmental sub-elements before and after applying the transitivity rule. CP: Circular Practices; FD: Facilities Design; SP: Sustainable Procurement; WRM: Waste Reduction & Management

Environmental sub-elements	CP	FD	SP	WRM	Priorities
Before applying the transitivity rule					
CP	1	5.6945	6.6207	6.0633	0.6074
FD	0.1756	1	6.195036	6.1743	0.2538
SP	0.1510	0.1614	1	0.1656	0.0393
WRM	0.1649	0.1619	6.0387	1	0.0995
After applying the transitivity rule					
CP	1	6	6	7	0.6774
FD	0.1667	1	1	1.1667	0.1129
WRM	0.1667	1	1	1.1667	0.1129
SP	0.1429	0.8571	0.8571	1	0.0968

Table 5 represents a pairwise comparison of social sub-categories, i.e., Patient Satisfaction, Employee Satisfaction, Affordability, and Sustainable Health, before and after applying the transitivity rule. In this case, the initial CR was 0.4233, which is why the transitivity rule was also applied here. Patient Satisfaction is not only the highest priority in the social sustainability factor in the healthcare sector, but it represents most of the priority in the social element, i.e., about 70%. Employee Satisfaction, Affordability, and Sustainable Health are all given the same importance after applying the transitivity rule, with each representing only 10% of the priority in the social element.

**Table 5:** Pairwise comparisons of social sub-elements before and after applying the transitivity rule. PS: Patient Satisfaction; ES: Employee Satisfaction; AF: Affordability; SH: Sustainable Health

Social sub-elements	PS	ES	AF	SH	Priorities
Before applying the transitivity rule					
PS	1	74.993	70.469	74.178	0.6412
ES	0.1334	1	75.271	77.506	0.2396
AF	0.1419	0.1329	1	77.219	0.088
SH	0.1348	0.1290	0.1295	1	0.0312
After applying the transitivity rule					
PS	1	7	7	7	0.7
ES	0.1429	1	1	1	0.1
AF	0.1429	1	1	1	0.1
SH	0.1429	1	1	1	0.1

The pairwise comparison multi-criteria function (after applying the transitivity rule) can be presented as the following:

$\begin{aligned} & \text {Multi-Criteria Utility Function} =0,05 *[\text {Green Growth}]+0,01 *[\text {Research and Innovations}]+0,01 \\ & *[\text {Savings in Operational Costs and Enhanced Profits}]+0,01 \\ & *[\text {Indigenous Production}]+0,32 *[\text {Circular Practices}]+0,05 \\ & *[\text {Facilities Design}]+0,05 *[\text {Waste Reduction and Management}] \\ & +0,05 *[\text {Sustainable Procurement}]+0,33 *[\text {Patient Satisfaction}] \\ & +0,05 *[\text {Employee Satisfaction}]+0,05 *[\text {Affordability}]+0,05 \\ & *[\text {Sustainable Health}] \end{aligned}$

**Figure 1:**
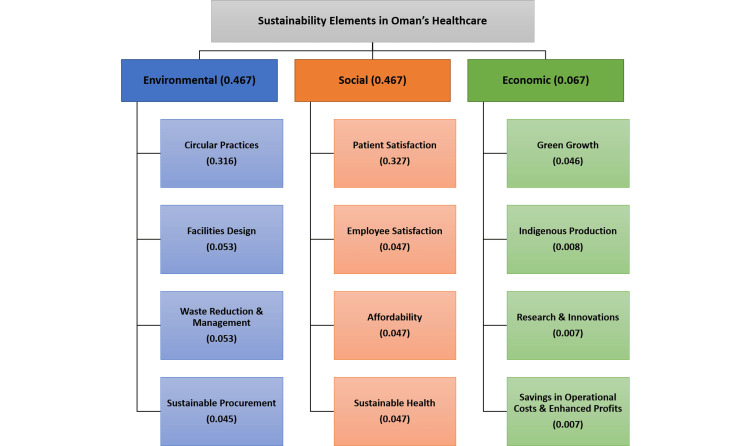
Sustainability elements in Oman’s healthcare with their relative importance.

**Figure 2:**
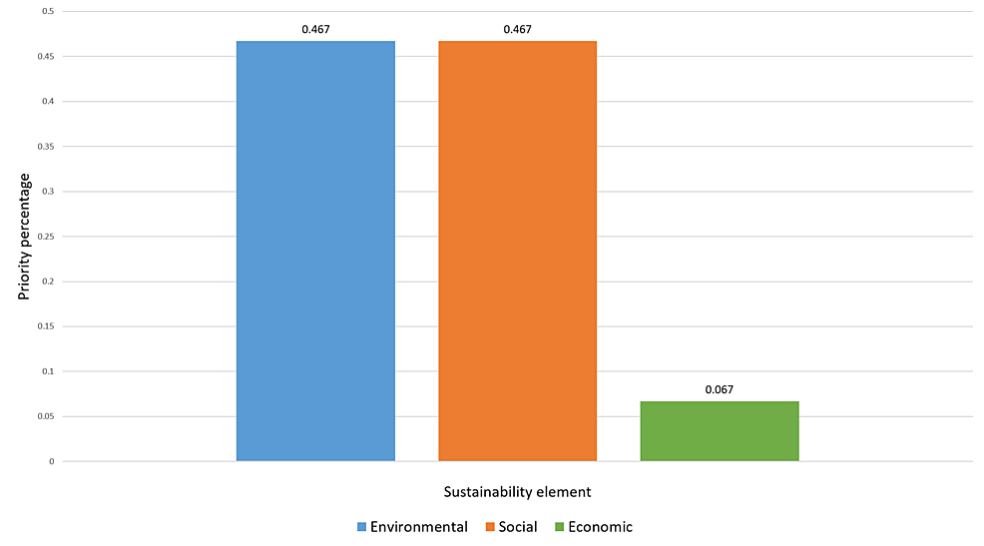
Priorities of the main sustainability elements in Oman’s healthcare sector.

Figure 1 represents the importance of each element and sub-category in sustainability factors in Oman’s healthcare. The ranking of the three main sustainability elements is illustrated in Figure 2.

**Figure 3:**
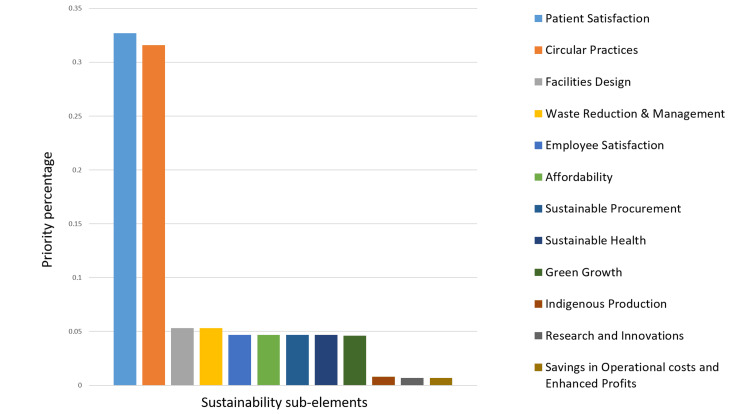
The overall ranking of the 12 sub-elements of healthcare sustainability in Oman.

Figure 3 shows that Patient Satisfaction and Circular Practices emerge as the highest-ranked priorities, with scores of 0.327 and 0.316, respectively. These indicate a strong emphasis on meeting patient needs and implementing sustainable practices. Conversely, Indigenous Production, Research and Innovations, and Savings in Operational Costs and Enhanced Profits are the lowest-ranked priorities, suggesting less immediate focus on these areas in comparison. In addition, Figure 3 shows the overall priority of the 12 sub-criteria of sustainability factors.

## Discussion

The AHP analysis examined the relative priorities of the environmental, social, and economic elements within Oman’s healthcare sustainability. Of the three main elements, environmental and social sustainability together represented 93.4% of the overall priority, outweighing economic sustainability. Despite the importance of the latter aspect for sustaining Omani healthcare, the results urge decision-makers to focus their efforts on environmental protection and social inclusion. Having such findings sheds light on how social and environmental aspects contribute to healthcare from a broader perspective.

Moving to the sub-element level, circular practices took the lead and accounted for 77.7% of the environmental priorities. This can guide healthcare operation managers toward fostering practices that allow resource recovery and reuse. It also affirms the need to approach healthcare holistically. Thus, it should go beyond care delivery to include procurement and waste management.

On the other hand, the analysis indicated that patient satisfaction reached the highest component in terms of societal sustainability, accounting for 70% of its elements’ priority. These outcomes emphasize the necessity of patient-centered care and an in-depth understanding of patients’ expectations, such as including open-ended questions, interactive communication during their appointments, engaging patients in the decision-making process, and attempting to adopt modern tools to reduce potential gaps, to eventually be more aware of their needs and preferences within healthcare delivery models.

Yet, it is necessary to clarify the limitations of the AHP methodology in this study. Although AHP offers valuable insights into stakeholder preferences, it relies on expert input and is intrinsically subjective. Furthermore, the research scope might be limited by elements such as sample size and the specific criteria used in the analysis. Nevertheless, by providing a relatively quantitative assessment of the importance of economic, environmental, and social factors associated with healthcare system sustainability, this study adds empirical evidence in addition to previous relevant research. It can contribute to decision-makers in the healthcare sector of Oman by adopting an integrated approach to healthcare sustainability and allocating resources to achieve this goal.

Comparing these findings with previous studies reveals both consistency and inconsistency. Kenny et al. [11] and Sherman et al. [12] showed differences regarding the prioritization of specific elements and sub-categories, which influence sustainability priorities across different healthcare systems. However, they agree with this study that environmental factors have a fundamental impact on achieving sustainable healthcare. In line with our findings, Klug et al. [13] emphasized the importance of setting sustainability priorities and proposing evaluation frameworks.

Furthermore, Mehra and Sharma [7] highlighted the significance of research and innovation in achieving sustainable healthcare. They emphasized the importance of circular practices, waste reduction, and management. In addition to Ossebaard and Lachman [14], who noted the urgency of addressing environmental sustainability in healthcare, they advocated for integrated responses to mitigate the environmental impact of healthcare services. On the other hand, Rattan et al. [15] prioritized sustainability dimensions differently from the literature review. While our study focused on economic, social, and environmental sustainability, the literature review emphasizes the environmental impact of healthcare activities and the need for standardized metrics.

Sustainability, as Rawat et al. [16] addressed in their study applying AHP, was the main aspect focusing on different disciplines, and we used it specifically to measure the priorities and challenges in the sustainability of the healthcare sector. Moreover, our study revealed that the three aspects of healthcare sustainability need to be dealt with holistically to achieve consistency for the upcoming generation and that stakeholders need to pay attention to improvement considering all dimensions.

One example that shows the readiness of Omani healthcare to deal with challenges is what Sohal et al. [17] found in their study when assessing readiness for lean thinking in healthcare settings in Oman. They focused on interviewing various stakeholders, highlighting the strong leadership support in Oman, understanding its value, and lean implementation by conducting the necessary training. Lean application in the Omani healthcare sector, despite the known challenges in strategic agendas and measurement systems, is highly encouraged. The authors suggested that the Omani healthcare system is empowered to apply lean processes in multiple sectors as awareness has already spread, which undoubtedly yields significant improvements.

Oman’s tertiary hospitals’ quality of care and patient safety were explored by Al-Jabri et al. [18], who studied the viewpoints of both patients and healthcare professionals. They reported a significant insight among healthcare professionals, slightly higher than among patients. Moreover, they found an association between hospital variables, overall quality of care, and patient safety among healthcare professionals. This indicates the crucial role of organizational factors in delivering quality healthcare services.

The impact of healthcare public expenditure on health outcomes in the Arab Gulf region, including Oman, was explored by Al-Azri et al. [19]. Their study revealed that healthcare expenditure has a noticeable impact on improving health outcomes, especially in reducing infant, child, and maternal mortality rates. In contrast, they found no significant impact on reducing deaths due to non-communicable diseases, which suggests the need for further investment and intervention. In general, these studies aid in a better understanding of Omani healthcare, highlighting its strengths and areas for improvement to deliver high-quality care with better effect.

The AHP analysis revealed that Oman’s healthcare is closely aligned with global healthcare trends and the growing recognition of the interconnection among healthcare, societal well-being, and environmental health. This is fully reflected in understanding the paradigm shift in the health sustainability process from a more comprehensive perspective that includes environmental protection and social inclusion. Therefore, the paradigm shift is considered one of the priorities for health sustainability. To justify such priority, parties must acknowledge the evolving landscape of healthcare, ensuring success measures for sustainability and well-being are completed by economic indicators. Oman’s healthcare system needs to prioritize environmental sustainability, especially circular practices, to reduce its environmental footprint and contribute to global efforts to combat climate change and environmental degradation. On the other hand, we cannot forget the focus on social sustainability by giving priority to patient satisfaction, which, in turn, greatly highlights the importance of patient-centered care and comprehensive healthcare practices in promoting equitable access and improving health outcomes.

Some explanations for the observed prioritizing can involve contextual, political, and cultural elements unique to the healthcare system in Oman. Stakeholder preferences for environmental and social sustainability over purely economic considerations may have been influenced by Oman’s demonstrated commitment to environmental stewardship and social development, as demonstrated by its involvement in global climate initiatives and investments in social welfare programs.

Literature on Oman’s healthcare sustainability is scarce. Thus, we believe this study has several theoretical and practical implications. Starting with the former, the present study expands the assessment tools for sustainability by using AHP. In other words, it allows for a deeper understanding of such a methodology and opens the possibility for further enhancement. Moreover, it reveals experts’ viewpoints on the relative importance of different sustainability aspects derived from long-standing experience in the field. The findings also show how these sustainability dimensions are integrated. Thus, they recommend seeking such a comprehensive approach for healthcare sustainability.

Regarding the practical implications, it highlights the necessity for government policymakers to promote environmental sustainability through programs such as green growth and circular practices. Furthermore, efforts must be deployed to enhance healthcare priorities and draw them into a reform agenda, with an emphasis on improving the model system for healthcare delivery, in addition to careful resource redistribution and allocation that aligns with the needs of each healthcare system.

Despite the valuable insights provided, this study has certain limitations. First, the analysis was based on subjective judgments inherent to the AHP methodology, which may introduce biases. Second, the study focused solely on priorities within the Omani healthcare context, limiting generalizability to other regions. Additionally, the study did not explore potential interactions between different sustainability elements, which could provide a more holistic understanding of healthcare sustainability priorities. Future research should aim to address these limitations by incorporating a broader range of stakeholders and perspectives, as well as employing complementary methodologies to validate and complement the findings.

Contradictions or surprises in the findings, if any, could prompt further investigation into the underlying factors shaping stakeholder preferences and priorities. For example, if economic considerations were unexpectedly low in priority, exploring the reasons behind this discrepancy could reveal insights into Oman’s unique healthcare context and stakeholder values.

Overall, this paper adds to the growing body of literature on healthcare sustainability as it reflects stakeholder preferences within Oman’s healthcare system. By highlighting the importance of environmental and social sustainability, as evidenced by the prioritization of circular practices and patient satisfaction, this study contributes to ongoing efforts to build resilient and equitable healthcare systems that meet the needs of both current and future generations.

## Conclusions

The findings of this research, based on AHP analysis, emphasize the higher weight of environmental and social sustainability over economic factors in Oman’s healthcare system. Circular practices had a key priority within the environmental domain, while patient satisfaction stood out within the social domain. The study highlights the need for policymakers and practitioners to prioritize investments and interventions that promote environmental protection and social inclusion within Oman’s healthcare system. Moving forward, future research could explore potential interactions between different sustainability elements and address limitations related to sample size and the specific criteria included in the analysis. Thus, contributing to a more comprehensive understanding of healthcare sustainability priorities and informing targeted interventions to enhance the resilience and equity of healthcare systems.
